# Assessment of thoracic ultrasound in complementary diagnosis and in follow up of community-acquired pneumonia (cap)

**DOI:** 10.1186/s12880-017-0225-5

**Published:** 2017-08-31

**Authors:** Maria D’Amato, Gaetano Rea, Vincenzo Carnevale, Maria Arcangela Grimaldi, Anna Rita Saponara, Eric Rosenthal, Michele Maria Maggi, Lucia Dimitri, Marco Sperandeo

**Affiliations:** 1 Department of Pneumology, “Federico II University”, AO “Dei Colli” Monaldi Hospital, Via Domenico Fontana,134, Naples, Italy; 2 Department of Radiology, AO “Dei Colli” Monaldi Hospital, Naples, Italy; 3 Unit of Internal Medicine, “Casa Sollievo della Sofferenza” Hospital, IRCCS, San Giovanni Rotondo (FG), Italy; 4 Unit of Internal Medicine and Pneumology, “Casa Sollievo della Sofferenza” Hospital, IRCCS, San Giovanni Rotondo (FG), Italy; 5 Unit of Internal Medicine, Local Health Service, Potenza, Italy; 6 Department of Internal Medicine, Hospital Archet 1, Nice, France; 7 Unit of Emergency Medicine, “Casa Sollievo della Sofferenza” Hospital, IRCCS, San Giovanni Rotondo (FG), Italy; 8 Unit of Pathology, “Casa Sollievo della Sofferenza” Hospital, IRCCS, San Giovanni Rotondo (FG), Italy; 9 Unit of Interventional and Diagnostic Ultrasound of Internal Medicine, “Casa Sollievo della Sofferenza” Hospital, IRCCS, San Giovanni Rotondo (FG), Italy

**Keywords:** Community acquired pneumonia (CAP), Thoracic ultrasound (TUS), Complementary diagnostic tool, Follow-up

## Abstract

**Background:**

Chest X-ray (CXR) is the primary diagnostic tool for community-acquired pneumonia (CAP). Some authors recently proposed that thoracic ultrasound (TUS) could valuably flank or even reliably substitute CXR in the diagnosis and follow-up of CAP. We investigated the clinical utility of TUS in a large sample of patients with CAP, to challenge the hypothesis that it may be a substitute for CXR.

**Methods:**

Out of 645 consecutive patients with a CXR-confirmed CAP diagnosed in the emergency room of our hospital over a three-years period, 510 were subsequently admitted to our department of Internal Medicine. These patients were evaluated by TUS by a well-trained operator who was blinded of the initial diagnosis. TUS scans were performed both at admission and repeated at day 4-6th and 9-14th during stay.

**Results:**

TUS identified 375/510 (73.5%) of CXR-confirmed lesions, mostly located in posterior-basal or mid-thoracic areas of the lungs. Pleural effusion was detected in 26.9% of patients by CXR and in 30.4% by TUS. TUS documented the change in size of the consolidated areas as follows: 6.3 ± 3.4 cm at time 0, 2.5 ± 1.8 at 4-6 d, 0.9 ± 1.4 at 9-14 d. Out of the 12 patients with delayed CAP healing, 7 of them turned out to have lung cancer.

**Conclusions:**

TUS allowed to detect lung consolidations in over 70% of patients with CXR-confirmed CAP, but it gave false negative results in 26.5% of cases. Our longitudinal results confirm the role of TUS in the follow-up of detectable lesions. Thus, TUS should be regarded as a complementary and monitoring tool in pneumonia, instead of a primary imaging modality.

## Background

Community-acquired pneumonia (CAP) is one of the most common infectious diseases contributing to mortality and morbidity worldwide [1]. Pneumonia exhibits a broad range of severity and induces many diagnostic and therapeutic challenges [2]. According to the American College of Radiology Appropriateness Criteria Expert Panel on Thoracic Radiology, a chest X-ray (CXR) is usually appropriate in patients with positive physical examination or risk factors for pneumonia [2, 3]. Standard CXR can identify pneumonia in almost all areas of the lung, and also helps to define its severity (multilobar or not) and the presence of complications, such as cavitations [4], and co-morbidity (intrathoracic diseases of the mediastinum and the heart). Computed Tomography (CT) is the golden standard for CAP diagnosis. However, it has a more limited role in daily clinical practice and is mostly used in dubious cases or in the assessment of complicated pneumonia, due to its higher cost and radiation exposure [2].

Besides these traditional diagnostic tools, thoracic ultrasound (TUS) is gaining growing popularity as a possible complementary tool for the diagnosis of pulmonary diseases. Some authors even went so far to say that TUS could represent an alternative tool for the diagnosis of pulmonary diseases, due to its intrinsic characteristics. TUS is a non-invasive and radiation free method, readily available in many clinical departments and are also suitable for bedside exam in critical care settings. Moreover, several studies by our and other groups showed that TUS may provide useful information in different pleural-pulmonary diseases [5, 6] and particularly in CAP [7]. Some authors even proposed TUS a possible substitute for CXR for the diagnosis of CAP [8], at least in selected groups of patients as pregnant women, children, or whenever radiation exposure should be limited. However, TUS cannot visualize foci of pneumonia which are not adherent to the pleural surface or positioned where ultrasound cannot penetrate. It should also be stressed that until now the current evidence-based guidelines on pneumonia diagnosis and management do not include TUS [9]. On the other hand, the TUS method could have a relevant complementary role in CAP diagnosis and management. Considering that the roles of several putative biomarkers in the management of patients with pneumonia are not definite, and can not provide clues for the occurrence of supervening complications [3, 4, 9–11]**.** In this sense, TUS could be an option to monitor the evolution of pneumonia foci following a CXR-confirmed diagnosis [12]. Despite the mentioned results and the growing number of studies on this matter, the debate on the role of TUS in the diagnosis and management of CAP is still ongoing.

Our study was aimed to investigate the clinical performance of TUS in the primary diagnosis and in the management of CAP, as compared to standard CXR. We evaluated the following aspects: a) how many cases of CAP were confirmed by TUS after clinical and CXR diagnosis, and b) how changes in TUS imaging appearances, from onset to recovery of CAP, could help identifying therapy failure or the need to investigate an alternative diagnosis.

## Methods

### Patients

We investigated all patients consecutively admitted to our department of Internal Medicine between September 2013 and November 2016 with a CXR-confirmed diagnosis of CAP. In the Emergency Room (ER) all patients had undergone a conventional diagnostic work-up, including anamnesis, physical examination, laboratory tests and chest radiography. The diagnosis of CAP was posed according to the American Thoracic Society (ATS) guidelines [13]. The CURB65 score [14] was used to drive the allocation of patients as follows: severe cases, with 3 or 4 criteria were referred to the intensive care unit –ICU- or to our Internal Medicine department; non-severe cases, at moderate risk, with 1 or 2 criteria were referred to ward or management as outpatients; non-severe cases, at low risk and with 0 criteria, were not hospitalized [15]. All patients timely received empirical therapy, according to guidelines for the evaluation and treatment of CAP. Patients with either contingent constraints or clinical conditions averting a complete TUS scan were conservatively excluded.

All participants gave witnessed informed consent and the study was approved by the ethics committee of SUN-AO dei Colli- Naples-Italy.

### Thoracic ultrasound examination

In all patients admitted to our department, TUS was performed by a blinded operator, who was not aware of CXR results, nor of the entire clinical-laboratory picture. In order to follow the evolution of CAP foci after therapy, TUS was performed in at least three repeated sittings: on day 0 (initial), between days 4 and 6 (intermediate), and between days 9 and 14 (final), according to a defined work-up [12] (see Table 1 for details. TUS was performed at the bedside by a physician with at least 10 years of ultrasound experience. An Esaote Technos MPX, Twice and My Lab30 Gold and Twice device (Genoa, Italy) using a multi-frequency (3.5–5 MHz and 3–8 MHz) convex probe and the pre-setting for thoracic ultrasound in B mode was used (depth of images penetration: 7–14 cm; gain control: 40-50%; use of harmonic imaging; electronic focus: pleural line). Each TUS was assessed for the number, location, shape, size, and breath-dependent changes of the consolidation area attributable to pneumonia. Two main sonographic patterns of lung consolidation were defined: hypoechoic consolidation and mixed consolidation (hypoechoic and hyperechoic). The dimensions of the consolidated areas are reported as the average between longitudinal and transversal axes. Local and basal pleural effusions were also recorded. In addition, the presence of spot and/or linear/arborescent hyperechoic images on TUS, improperly referred to as an air bronchogram, were also recorded. The presence of artefacts (increased TUS B-line counts in the hemithorax with consolidation) was not considered in this study, because such artefacts are at best a sensitive, but very non-specific sign of lung injury, common to many conditions [16, 17].

**Table 1:** TUS procedures

• Pulmonary thoracic assessment setting (including: tissue harmonics imaging activation,the time gain compensation (TGC) should not exceed 50%, electronic imaging focus on the pleural line) using mainly a 3.5-5 MHz convex probe EsaoteTechnosMpx**,** My Lab 30 and Twice (Genova, Italy).
• Patients’ chests were examined posteriorly, lateral and anteriorly, while sitting. A few patients were examined in a semi-supine position, due to severe discomfort when sitting upright. Posteriorly, we opted for longitudinal and transversal interscapular and paravertebral line scans. Anteriorly, the longitudinal and transversal interclavicular, parasternal line and supraclavicular scans were used.Laterally, we used the longitudinal and transversal anterior, median and posterior line axillary views.
• The duration of ultrasound probe application in each site (posterior, lateral and anterior) was 4–5 min and overall time needed to complete the entire lung examination was 12–15 min.

The positive clinical evolution of CAP was detected by clinical assessment and CXR, and faced to the changes of TUS findings during stay and/or within 30 days on an outpatient basis after discharge.

### Assessment of inter-reader agreement

Video-clips recorded during TUS examinations (each lasting a minimum of 3 min) were later reviewed by a second examiner, who was blinded of all previous TUS findings; clips for control assessment were randomly assigned to one of two examiners with 20 years of experience in transthoracic ultrasound.

### Statistical analysis

The results concerning the dimensions of TUS-detectable lesions are presented both as range and as mean ± SD. Inter-reader agreement was assessed using Spearman’s coefficient for all parameters. The significance of changes in size of US-detectable lesions over time was tested by Repeated Measures ANOVA. A *p* value of <0.05 was considered significant.

In the clinical application phase of the study, a repeated measurements ANOVA model (on basal, 4th day, and 8–10th day) was used to assess dimensional changes over time in lung consolidation areas and was carried out via linear mixed models. Within-patients correlation was accounted for by an unstructured correlation type matrix [18]. Hochberg’s method was followed to obtain *p*-values corrected for multiple comparisons. *P*-values <0.05 were considered significant. All analyses were performed using SAS Release 9.1 (SAS Institute, Cary, NC, USA). Inter-reader agreement was assessed using Spearman’s coefficient for all parameters.

## Results

### Patients

**Fig. 1:**
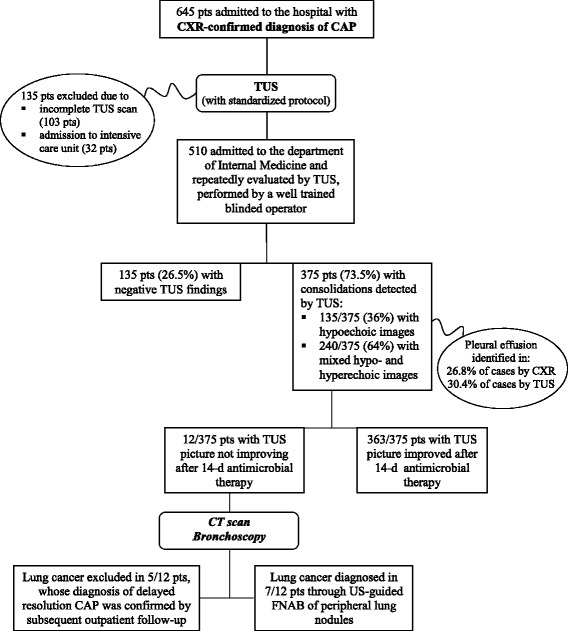
Flow-chart of the main results

Seven hundred ninety-six consecutive adult patients presented to the emergency room of the “Casa Sollievo della Sofferenza” Hospital (San Giovanni Rotondo - Italy) with symptoms and clinical/laboratory signs consistent with the diagnosis of CAP. Following the conventional diagnostic work-up, in 736 of them the diagnosis was confirmed by chest X-ray (CXR), and 91 patients were discharged and managed as outpatients after the ER workup. Among the 645 patients admitted to the hospital, 32 were referred to the intensive care unit, and 613 to our department. Among the latter, 103 patients were excluded (see methods). Five hundred ten patients admitted to our department were finally studied (see Fig. 1). The demographic and clinical characteristics of investigated patients are reported in Table 2.

**Table 2:** Characteristics of the included patients (*n* = 510)

Age (years), (mean ± SD)	Range 32-78 (58.4 ± 14.7)
Gender (M⁄F)	281/229
CURB 65	2.4 ± 0.6
Mean hospital stay	8.9 ± 2.5 days
Consolidated areas identified by TUS	375/510
Size of Consolidated areas (cm)	6.3 ± 3.4
Comorbidity(more than one in 60 pts)	455/510 pts.(89.2%)
Diabetes mellitus	96 (18.8%)
COPD	107 (21%)
Pulmonary fibrosis	28 (5.5%)
Heart failure (III-IV NYA)	80 (15.7%)
Chronic kidney diseases	12(2.3%)
Oncological diseases	68 (13.3%)
Coronary disease	56(11%)

### Initial TUS findings

**Fig. 2:**
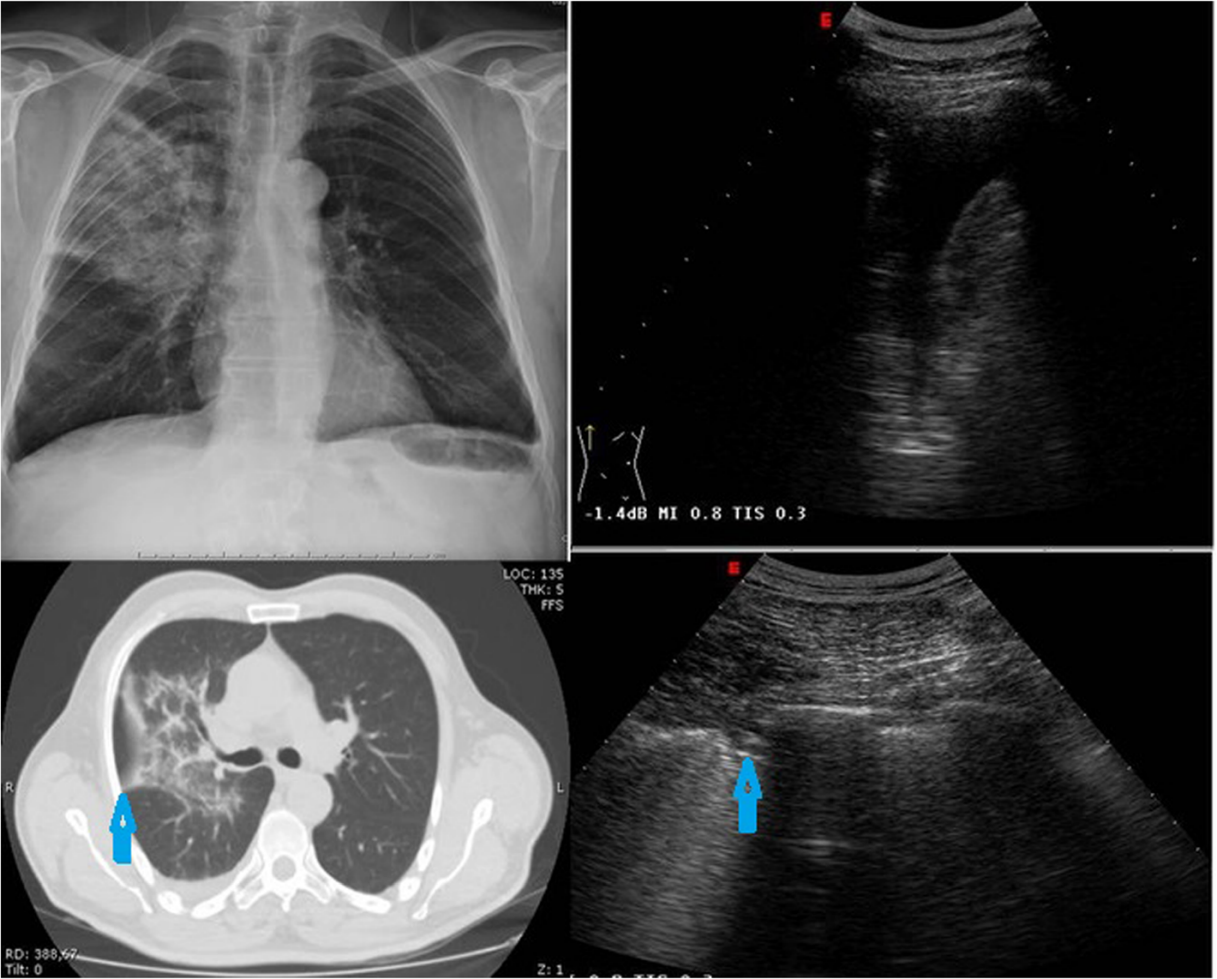
Right lobar pneumonia. Lung consolidation is well defined by CXR (top left) and CT (bottom left); by TUS, pleural effusion is easily identified (top right) but only a very small density and adherent to pleura pneumonia is visible (blue arrows) (bottom right)

**Fig. 3:**
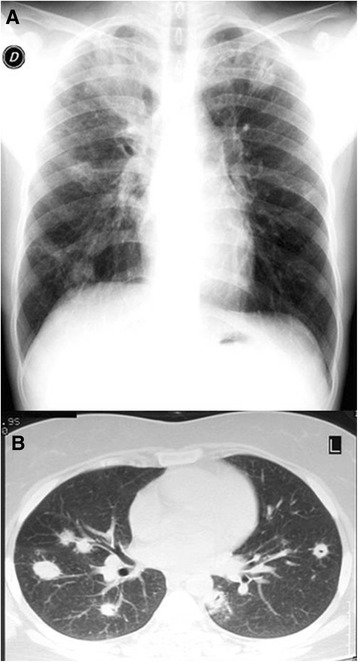
**a** Multifocal pneumonitis in an immunocompromised patient. **b** Disease was not strictly subpleural, and TUS was not contributory

The topographic distribution of lung consolidation detected by TUS is indicated in Table 3. TUS imaging was negative for consolidation attributable to pneumonia in 135 adults with CXR-recorded pneumonia, which implies a false negative rate of 26.5%. This was due in 72/135 patients to single consolidations that were neither sub-pleural nor retro-scapular, and in 63/135 patients to multiple, even bilateral, and mostly not strictly sub-pleural, consolidations, which were visible only minimally using TUS (Fig. 2). Of the latter group, 39 patients were subsequently found to be immuno-compromised (Fig. 3).

**Table 3:** Topographic distribution detected at lung ultrasound examination of pneumonia patients (*n* = 375)

Localization of pulmonary focus	Number of patients (*n* = 375)
Posterior-basal	202 (54%)
Posterior mid-thoracic	60 (16%)
Posterior-lateral mid-thoracic	75 (20%)
Anterior mid-thoracic	15 (4%)
Para-cardiac	12 (3.2%)
Anterior apical	6 (1.6%)
Posterior apical	3 (0.8%)
Multiple consolidation	31 (8.3%)

Among US-detectable lesions (found in 375/510 pts. = 73.5%), most were posterior basal o midthoracic (see Table 3). Maximal length of the consolidation area ranged from 3.5 to 9.5 cm (mean ± SD: 6.3 ± 3.4 cm). The CXR features varied from complete lobar consolidation to patchy or less severe opacity, whereas TUS imaging showed two main patterns: hypoechoic in 135/375 (36%) and mixed in 240/375 (64%).

Pleural effusion associated to detectable consolidation was detected by TUS in 114⁄375 (30.4%) patients and in 137⁄510 (26.9%) patients by CXR. Spots, stripes and/or linear/arborescent hyperechoic images were present in 54.9% of all patients (206/375), and no difference was observed in prevalence between genders, or specific association with disease severity or greater dimension of consolidation.

### Treatment-induced changes of TUS findings

By monitoring lung consolidations in subjects with moderate risk CAP using a convex probe (3.5-5 MHz), TUS modifications were: initial dimensions 3.5 to 9.5 cm (6.3 ± 3.4), intermediate dimensions 2.1 to 4.3 cm (2.5 ± 1.8 cm); final dimensions 0.3 to 1.0 cm (0.9 ± 1.4 cm) (*p* < 0.001 by ANOVA). A favourable outcome was confirmed in all except 12 patients by a final normal CXR and/or a subsequent clinical assessment including normal CXR and TUS within the first month, together with complete disappearance of fever and of the most relevant symptoms and physical signs. A persistent localized pleural line thickening (> 3.0 mm with 3.5 MHz convex transducer) after resolution was observed in all pneumonia patients. In 12/375 (3.2%) patients (three women and nine men) pulmonary consolidation, diameter 4.5 to 5.5 cm, did not significantly improve despite intensive antibacterial therapy, with no satisfactory clinical improvement or resolution of fever. In all 12, a chest CT was performed: in five patients the diagnosis of pneumonia was confirmed, and all recovered, although with delay. In the other seven patients, the diagnosis of lung cancer suggested by chest CT was subsequently confirmed at histology on US-guided fine needle aspiration biopsy (FNAB) of the lung nodules. In these cases, previous bronchoscopy did not provide any diagnostic yield, conceivably because of the peripheral site of the lesions. The spot and linear hyperechoic images were observed in 5/7 of the patients with a subsequent diagnosis of lung cancer.

### Inter-reader agreement

Inter-reader agreement was excellent (Spearman’s coefficient ≥ 0.90 for all parameters).

### Positive TUS findings in patients with negative CXR imaging

Pneumonia that was not preliminarily CXR-proven but was suggested by the clinical picture and by the finding of TUS areas attributable to consolidation was identified in ten patients. They showed small sub-pleural consolidation areas of 1.0 to 1.5 cm; these cases did not progress toward greater extension of consolidation, and those considered doubtful for pneumonia were excluded from the subsequent data analysis of TUS imaging distribution.

## Discussion

Our current results, obtained from the largest ever investigated series, substantially confirm the previous ones we obtained from an independent series of inpatients [12]. In most cases of CAP, TUS examination detects the sites of inflammation, which have typical although not specific patterns. TUS allows the measurement of dimensions of the pulmonary focus before and after medical therapy, which implies the possible use of this tool to monitor treatment efficacy. The present data also confirm the higher sensibility of TUS in identifying pleural effusion and its role to facilitate fluid drainage. Accordingly, in US-detectable cases TUS appears to valuably integrate the diagnostic information obtained from a CXR alone [19]. Due to these and other reasons, several authors went so far to even suggest that CXR can be replaced by TUS in the clinic to identify CAP [20, 21]. However, our current results mandate extreme caution on this matter.

Actually, most cases of CAP (around 80% of cases) are subpleural (that is adherent to the 70% of pleura visualized by TUS) and begin peripherally. This explains why CAP is also most often visible by TUS [13, 22]. In our series, 73.5% of CXR-positive lung consolidations due to CAP were also visible with TUS, being localized in subpleural areas (see details on location in Table 3). In these sites, hypoechoic and mixed (hypoechoic and hyperechoic) lesions corresponded to the foci of pneumonia identified by CXR. This result confirms in a large cohort of patients the reliability of TUS imaging to corroborate the diagnosis of CAP obtained from CXR. However, it should also be stressed as more than one out of four cases of CAP (26.5%) were not detectable by TUS, even if performed with the highest methodological accuracy (standardized complete scan, technical accuracy, involvement of only highly experienced operators, and so on) [23]. Several reasons underpin this high false positive rate. As a fact, TUS cannot visualize foci of pneumonia which are not adherent to the pleural surface or are positioned where ultrasound cannot penetrate (e.g. adjacent to the mediastinum) [24–26]. Moreover, TUS is not a valid aid in immunocompromised patients of intensive care units [27] who commonly suffer from severe *Staphylococcus*, *Aspergillus*, *Candida*, *Mycoplasma* and viral pneumonia. Such pneumonia foci are often intra-parenchymal, multiple, and/or outside the TUS-visible pleura**.** This is also indirectly supported by our current findings, since most cases of CXR-confirmed CAP we did not detect by TUS had insufficient pleural contact and were also clinically more severe, as is usually observed in immunocompromised patients [24]. As a consequence, performing TUS alone, many pneumonias which would have been detected by CXR, can remain undiagnosed. Indeed, TUS and CXR examine lungs in different ways, with only a partial overlap.

In adjunct, it should be stressed as, even in US-amenable areas, TUS findings as spots, stripes and/or linear/arborescent hyperechoic images (improperly called air bronchograms) are not disease-specific. Therefore, TUS imaging is not helpful to differentiate between pneumonia and other lung diseases [17], including cancer [17, 25]. In particular, no study or meta-analysis so far demonstrated that linear/arborescent hyperechoic images on TUS do really correspond to the CT imaging finding of air bronchogram [22]. As a matter of fact, noteworthy, we also found this US feature also in 5/7 patients finally diagnosed to have lung cancer. This latter finding definitely confirms as certain optimistic statements on this matter are not realistic at all, CT remains the gold standard for imaging diagnosis. Moreover, according to our previous experience, we deliberately chose not to include the evaluation of B-line or “ring-down” artefacts among the investigated parameters. Despite their wide popularity, these TUS signs may be found in patients with different conditions, because these artefacts are generated behind the pleural line because of the high difference of acoustic impedance between soft tissue and air, or between fluid and air. Such a difference is enhanced in a number of pleuro-pulmonary diseases [18]. As a consequence, the attention to this acoustic phenomenon could be highly misleading in patients with co-morbidities, as were most patients from our series and in the common clinical practice.

On the other hand, our results stressed as TUS monitoring allows for follow-up care after the preliminary clinical-radiological diagnosis of pneumonia [4, 11–13], being capable to demonstrate the decrease in size of consolidation. This could be a precious clinical information, since the management of patients with CAP who fail to improve constitutes a relevant challenge for clinicians. Changes in CRP levels for CAP patients are sufficiently useful to discriminate between true treatment failure and slow response to treatment and can help clinicians in management decisions when patients fail to improve [28, 29]. CT should be performed to help rule out lung cancer when there is a lack of dimensional reduction of consolidations, and/or failure of symptom regression. Under these conditions and according to our results, TUS can be used to provide bedside information on the persistence of lung consolidation and can be a useful adjunctive tool to check the response to treatment [29–32]. The late observation of persistent localized pleural-line thickening after resolution is seemingly only the signature of the recent pneumonia. Our data suggest a wider use of TUS in the follow-up after the initial CXR diagnosis of sub-pleural pneumonia, whose progressive reduction in size is reliably assessed. Accordingly, TUS could also decrease the need to repeat radiological procedures, particularly in the follow-up of pregnant patients or in the follow-up of patients not requiring hospital admission. When scheduling follow-up on an outpatient basis, TUS is seemingly a less expensive procedure and it is already successfully used to monitor other conditions and diseases [33].

Discordant results have been reported on TUS-positive and CXR–negative by other authors [2, 3, 13, 22]. Such discrepancies may result from Rx–negative small lung consolidations detected exclusively on TUS. Alternatively, they may stem from the different relationship between lung consolidations and the pleura, or from an alternative diagnosis (not CAP), or from the presence of sub-segmental lung focal areas of atelectasis beyond terminal bronchioles [23]**.**

Our study has several strengths. Firstly, we prospectively validated our previous results by studying an independent large series of unselected patients. All patients of our series were managed in a substantially coherent way, at variance with previous studies suffering from a wide variability in criteria of admission [31], management, and discharge of pneumonia patients without follow up.

Our study has some limits, too. In fact, we tried to mimic the common practice through the unselective inclusion of all patients coming to the emergency room of our hospital and being suitable for subsequent (repeated) observation in our Internal Medicine department. Such a design was aimed to reduce possible observational bias, but this way we excluded patients with an insufficient number of TUS assessments, which implied the exclusion of patients admitted to other units, including ICU. Accordingly, the number of recruited patients with more severe disease and with likely more significant co-morbidities and further complications was lower. On the other hand, the exclusion of those managed as outpatients also excluded less severe cases. In addition, all TUS were performed by a highly trained staff and this could undermine the generalizability of our results. Actually, TUS requires a technically experienced operator and appropriate machine settings [11, 12, 30]. The clinical assessment of TUS consolidation mostly depends on the subjective expertise of the ultrasound operator, as in most sonographic diagnoses. Interpretation of TUS is not the easiest component of any ultrasound course and has many pitfalls, mostly for false-negative results. This is a risk increased by over-confidence [21]. As a fact, the negative ethical and potential medico-legal implications of omitting a CXR (co-morbidity associated, intraparenchimal not subpleural consolidation and therefore incorrect or incomplete diagnosis), particularly when addressing the therapeutic choices, are evident [34].

## Conclusion

In conclusion, we exclude that TUS could reliably replace CXR, and we confirm that the assessment of physical signs, CXR, and biomarkers such as procalcitonin and CRP, remain the pillars for the diagnosis of pneumonia. On the other hand, TUS represents a highly valuable complementary imaging procedure, which can be performed at bedside, and easily repeated after the initial assessment. Therefore we recommend its use as a complementary and monitoring tool.
